# Impact of Unilateral Orbital Radiotherapy on the Structure and Function of Bilateral Human Meibomian Gland

**DOI:** 10.1155/2018/9308649

**Published:** 2018-10-25

**Authors:** Di Chen, Xiaowei Liu, Ying Li, Hui Guan, Fuquan Zhang

**Affiliations:** ^1^Department of Ophthalmology, Peking Union Medical College Hospital, Chinese Academy of Medical Sciences & Peking Union Medical College, Beijing 100005, China; ^2^Department of Radiotherapy, Peking Union Medical College Hospital, Chinese Academy of Medical Sciences & Peking Union Medical College, Beijing 100005, China

## Abstract

**Background:**

Radiotherapy (RT) has widely been used to treat ocular tumors, yet the impact of orbital radiation to the meibomian gland has rarely been studied. Our study aims at evaluating the bilateral meibomian gland structure and function 12 months after unilateral orbital RT in patients with ocular tumors.

**Methods:**

An observational case-control study. A total of 10 eyes 12 months after unilateral orbital RT, 10 contralateral eyes, and 10 normal controls were enrolled. Meibomian gland loss (MGL), lipid layer thickness (LLT), tear film breakup time (TBUT), Schirmer I test, and cornea fluorescein staining were measured. Ocular Surface Disease Index (OSDI) of the RT patients was assessed and compared with normal controls.

**Results:**

The cumulative median radiotherapy dosage for the patients was 45 (range: 30, 70) Gy. The OSDI score of the patients was significantly greater than the normal controls (22.92 (range: 10.42, 37.50) vs 6.25 (range: 2.08, 10.42), *p* ≤ 0.001). Significant differences of upper MGL, lower MGL, LLT, and TBUT were found between the diseased eyes and normal controls (37.79% (range: 12.87, 92.41) vs 12.63% (range: 6.13, 42.34), *p*=0.007; 61.31% (range: 44.67, 87.98) vs 15.53% (range: 7.65, 45.13), *p* ≤ 0.001; 40 ICU (range: 23, 100) vs 81.5 ICU (range: 54, 100), *p*=0.007; 3.5 s (range: 2, 8) vs 6.5 s (range: 5, 10), *p*=0.002). The upper MGL and TBUT of the contralateral eyes were also considerably damaged compared with normal controls. Lower eyelid MGL and cornea staining score of the diseased eye were significantly correlated with radiation dosage (*r* = 0.913 and 0.680; *p*=0.001 and 0.044, respectively).

**Conclusion:**

Orbital radiotherapy could cause significant damage to the meibomian gland structure and function, not only the diseased eyes but also the contralateral eyes.

## 1. Background

Meibomian gland is a large sebaceous gland located in the eyelids and responsible for the lipid layer of tear film [1]. Damage to the meibomian gland could cause tear film instability, tear hyperosmolarity, and eventually evaporative dry eye, jeopardizing ocular surface health [2]. Multiple factors could affect meibomian gland function, including aging, deficiency of sex hormones notably androgens, other systemic conditions such as Sjogren's syndrome, psoriasis, and hypertension [3–6]. Besides, the meibomian gland is also vulnerable to external pathological factors due to its superficial location, such as trauma, ocular surgeries, and chemical burns [7–9]. However, the radiation damage of the meibomian gland has rarely been evaluated.

Radiation has been widely used to treat tumor patients, including ocular malignancies [10]. Even though there are effective shielding and precise radiation localization techniques available, orbital radiotherapy (RT) can still induce normal tissue damage and functional impairment, such as cataract, retinopathy, keratopathy, and dry eye [11, 12]. Our study aims at revealing the morphological and functional changes of the bilateral meibomian gland after unilateral orbital RT.

## 2. Methods

### 2.1. Study Population

This observational case-control study was conducted at the Department of Ophthalmology in the Peking Union Medical College Hospital. The patients after orbital RT and normal controls were enrolled between April 1, 2016, and March 31, 2017, in our facility. This study adhered to the tenets of the Declaration of Helsinki and was approved by the Institutional Review Board of Peking Union Medical College Hospital. Informed consents were obtained from all subjects.

The RT group included patients who were 12 months after the last session of unilateral orbital RT due to ocular tumors. Exclusion criteria included (1) patients aged less than 18 years old, (2) previous meibomian gland surgery or trauma, (3) meibomian gland or lacrimal gland carcinoma, and (4) bilateral orbital RT. Normal controls were sex- and age-matched participants who did not have any clinical signs or symptoms of ocular surface diseases and were not using any eye drops. Both eyes of the RT patients and the left eye of the normal controls were selected for evaluation.

### 2.2. Study Protocol

All subjects completed the Ocular Surface Disease Index (OSDI) questionnaire that contains 12 items and scores a range of 0 (no symptoms) to 100 (severe symptoms) points [13]. Clinical measurements were performed in the following order to minimize the effects of the previous test: (1) lipid layer thickness (LLT), (2) tear film breakup time (TBUT), (3) corneal fluorescein staining, (4) Schirmer I test, and (5) meibography.

### 2.3. Subject Examination

(1) LipiView® II Ocular Surface Interferometer (TearScience Inc, Morrisville, North Carolina, USA) was used to measure the LLT and take the meibography as described [14]. The unit of LLT is interferometry color units (ICU) based on the observed mean interference colors. (2) TBUT was measured 3 times consecutively under slit-lamp biomicroscopy after sodium fluorescein staining, and the median value was recorded. Fluorescein-impregnated paper strip (Tianjin Jingming New Technological Development Co, Ltd, China) was moisturized with saline and then gently applied to the tarsal plate of the lower eyelids for ocular surface staining. (3) Corneal staining was scored according to the NEI/industry grading system (range, 0–15) [15]. (4) Schirmer I test was observed for 5 minutes without anesthesia by a sterile Schirmer test strip (Tianjin Jingming New Technological Development Co, Ltd, China). (5) Meibomian gland loss (MGL, %) was defined as the ratio of the meibomian gland dropout area to the total area outlined by the polygon selection tool of ImageJ (1.47v, National Institutes of Health, Bethesda, Maryland, USA). The total area of the meibomian gland was defined as follows: the proximal border was estimated to be where the glands would have ended in normal MG morphology, the distal border was the actual ending of the glands, the nasal border was defined as the tear punctum, and the temporal border was defined to be the most visible tarsal conjunctiva of the everted lid [16]. All the examinations and MGL calculations were done by one experienced ophthalmologist (D. Chen) who did not know the patient previous medical history at the time of measurement.

### 2.4. Statistical Analysis

SPSS version 17.0 (SPSS, Inc, Chicago, Illinois, USA) was applied for the statistical analysis. Due to the small sample size of the study, all values were described as median (ranges), and nonparametric tests were used. The alpha level for all tests was 0.05, and the tests were two-tailed. The Mann–Whitney test was used for the comparison between the RT patients and the normal controls. The Wilcoxon rank test was used for the comparison between the diseased eyes and contralateral eyes. Spearman rank correlation analysis was applied to measure the degree of association between the RT dosage and ocular surface parameters. *p* value < 0.05 was considered statistically significant.

## 3. Results

### 3.1. Demographics

A total of thirty eyes (10 diseased eyes, 10 contralateral eyes, and 10 normal eyes) were enrolled. The median age of the RT and control group was 46 (range: 33, 79) and 46 (range: 35, 79) respectively (*p*=0.971). The sex distribution was identical in both groups (6 males, 4 females).

### 3.2. Radiotherapy Regimen of the Patients

The RT details are summarized in Table 1. All of these RT patients had biopsy-proven tumor diagnosis. Six out of the ten patients received RT due to stage IE conjunctival mucosa-associated lymphoid tissue lymphoma (MALToma), two due to ocular lymphoma, and the other two due to eyelid basal cell carcinoma. External-beam radiation was delivered in daily doses of 1.8 to 2.0 Gy, and the cumulative median RT dosage was 45 (range: 30, 70) Gy.

### 3.3. Comparison of Meibomian Gland Structure and Function between the RT Eye, Contralateral Eye, and Normal Controls

Both the upper and lower MGL of the diseased eyes were substantially greater than the contralateral eyes and normal controls. The LLT and TBUT of the diseased eyes were also significantly different than the normal controls (*p*=0.007  and  0.002, respectively) (Table 2). In addition to the diseased eyes, RT also caused significant changes of the upper meibomian gland and TBUT of the contralateral eyes compared with normal controls (both *p*=0.019). No significant differences of the Schirmer I test and cornea staining score were found among these groups. The OSDI score of the RT patients was significantly greater than the normal controls (22.92 (range: 10.42, 37.50) vs 6.25 (range: 2.08, 10.42), *p* ≤ 0.001).

**Figure 1:**
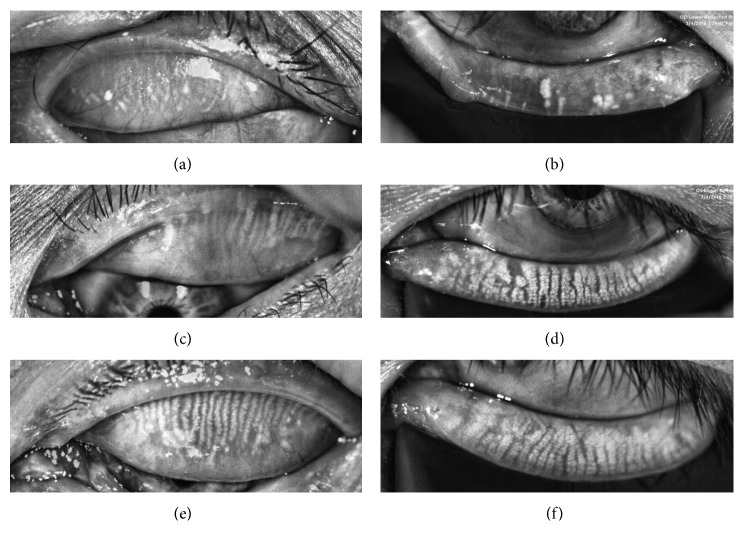
Bilateral meibomian gland loss 12 months after unilateral radiotherapy. Meibography shows the upper and lower meibomian gland loss in the diseased eye (a, b) and contralateral eye (c, d) of a 46-year-old male 12 months after unilateral radiotherapy due to right conjunctival MALToma and relatively normal meibomian gland of the left eye of a 52-year-old male control (e, f). MALToma: mucosa-associated lymphoid tissue lymphoma.

**Figure 2:**
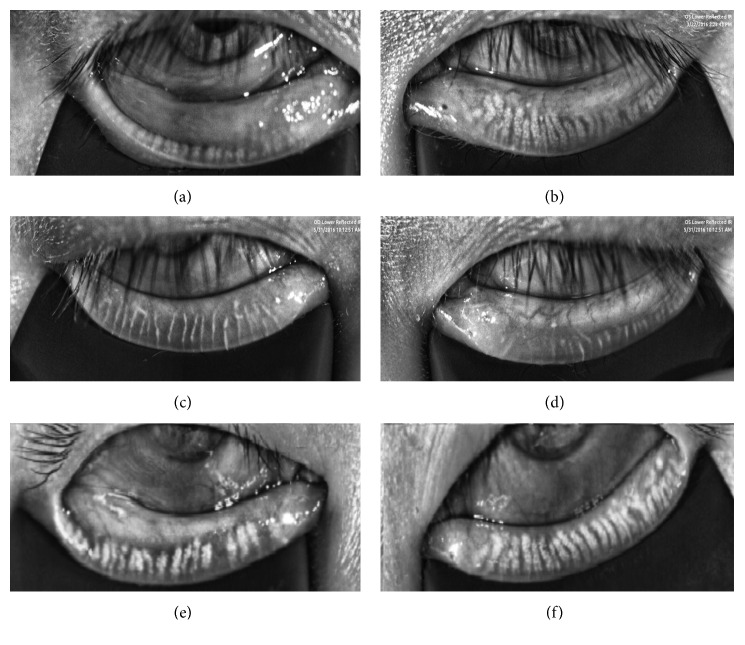
Acute and chronic changes of the bilateral lower meibomian gland after unilateral radiotherapy. Lower eyelid meibography of both eyes of a 33-year-old female with right conjunctival MALToma before (a, b), 3 days after (c, d), and 1 year (e, f) after right orbital radiotherapy. The changes of the meibomian gland structure of the diseased eye are shown in (a), (c), and (e), while the contralateral eye in (b), (d), and (f). Part of the meibography was blocked by the conjunctival tumor (∗) before radiotherapy (a). Prominent meibomian gland shrinkage was found 3 days after radiotherapy not only in the diseased eye (c) but also in the contralateral eye (d). Most of the meibomian gland structure recovered 12 months after radiotherapy with more prominent meibomian gland loss in the diseased eye (e) and mild meibomian gland loss in the contralateral eye (f). MALToma: mucosa-associated lymphoid tissue lymphoma.

Diffuse MGL was found in both eyelids of the diseased eye 12 months after RT (Figures 1(a) and 1(b)), compared with normal controls (Figures 1(e) and 1(f)). The upper and lower meibomian gland loss of the contralateral eyes were relatively mild with more obvious changes of the upper eyelid (Figures 1(c) and 1(d)). One patient (33-year-old female) with right conjunctival MALToma visited our clinic before and three days after RT. Part of the meibography of the diseased eye was blocked by conjunctival tumor before radiotherapy (Figure 2(a)), while the contralateral eye appeared quite normal then (Figure 2(b)). We noticed that the meibomian gland of her both eyes shrank to a linear configuration three days after RT (Figures 2(c) and 2(d)). However, most of the meibomian gland reappeared 12 months later with more prominent MGL in the diseased eye (Figure 2(e)) and mild MGL in the contralateral eye (Figure 2(f)).

### 3.4. Correlation of RT Dosage and MGL

Correlation analyses between RT dosage and ocular surface objective measurements are summarized in Table 3. The lower MGL of the diseased eyes showed a significant positive correlation with RT dosage (*p*=0.001), though no significant correlations were found between the MGL of the contralateral eyes with the RT dosage. In the diseased eye group, cornea surface staining also showed a significant positive correlation with RT dosage (*p*=0.044). However, this correlation was not found in the contralateral eyes either.

## 4. Discussion

Previous studies have revealed multiple side effects of orbital RT, including cataract, keratitis, macular edema, radio-induced retinopathy, and dry eye, yet rare has been reported about the toxicity of RT on the meibomian gland [10, 17, 18]. Karp et al. have reported meibomian gland atrophy induced by radiation through histological analysis, and one recently published study also discussed radiation-induced meibomian gland damage [19, 20]. However, our study compared bilateral meibomian gland damage with normal controls induced by unilateral RT. Besides, our study measured both upper and lower MGL and can serve as a complement to Woo's study which only assessed the lower MGL [20]. Also, since we noticed radiation-induced meibomian gland damage might change with time; our study evaluated patients who were exactly 12 months after RT, while Woo's study assessed patients with a wider range (3–70 months after RT) [20]. Such disparity of follow-up time might explain the different findings between our study and Woo's study.

The meibomian gland is a large holocrine sebaceous gland that requires constant renewal and differentiation of meibomian gland acinar cells [1, 21]. This means meibomian gland acinar cells are metabolically highly active, which makes them more sensitive to radiation. Besides, the meibomian gland is a superficial tissue located in the eyelid tarsal plate, and this location also makes it more vulnerable to radiation. Ionizing radiation could cause normal tissue injury through multiple mechanisms, involving the generation of reactive oxygen species and subsequent proinflammatory processes, innate immune responses, and DNA damage [22–25]. Radiation-induced long-lived free radicals are thought to cause progressive damage to normal tissues. More recent molecular studies suggest that depletion of tissue stem cells and progenitor cells by radiation could lead to much greater cell loss and tissue damage [26, 27].

Scattered radiation beams might explain the contralateral gland damage, especially the photon beams used in tomotherapy [28]. Upper MGL of the contralateral eyes was found significantly greater than normal controls, but not the lower MGL. Several studies have shown that lower MGL is more prominent than upper MGL in normal subjects [16, 29, 30]. This relative obvious MGL of the lower eyelid in normal population might attenuate the effect caused by RT. Besides, the relatively higher nose bridge adjacent the lower eyelid might block certain irradiative electrons to the lower meibomian gland. These changes suggest more effective protection measures that should be taken for the contralateral eyes during unilateral orbital RT.

We also noticed the linear configuration of the meibomian gland of both eyes three days after RT and its “recovery” 12 months later in one case. To our best knowledge, this phenomenon has never been reported before, and it might be explained by following mechanisms. The meibomian gland is embedded in tarsus connective tissue [31, 32]. The orbicularis muscle, located on the external side of the tarsal plate, generates compression against the meibomian gland and promotes the flow of meibum to the lid margin [1, 33]. Considering soft tissue edema is a common sequela of RT [34], and radiation might cause the edema of tarsus connective tissue and orbicularis muscle, thus compressing the meibomian gland to a linear configuration. As the edema gradually resolves with time, the meibomian gland might return to its “normal” appearance. Besides, the possibility of the meibomian gland acini regeneration cannot be excluded. Such meibomian gland reappearance might worth further exploring for the future stem cell research. The dramatic alteration of the meibomian gland in the contralateral eye further demonstrates that the radiation beam could affect the contralateral eyes, even though the RT is intended to be unilateral. It also implies the meibomian gland loss caused by RT might change with time.

The meibomian gland is the main source of lipid for the human tear film that prevents it against evaporation, and thus meibomian gland damage could impair tear film stability [1]. With the significant loss of the meibomian gland, the patients after RT showed significant shorter TBUT of both eyes and reported much higher OSDI scores than the controls. It has been proved that tear film characteristics are significantly correlated to MGL, and the lower eyelid meibomian gland may play a more vital role in it [3, 14, 35]. Thus, it is not surprising to find the significant decrease of LLT in the diseased eyes and no change in the contralateral eyes, considering the different impact of RT on the lower MGL between these two groups.

The effect of radiation on the meibomian gland seems to be dose-dependent, which indicates appropriate balance of the efficacy, and toxicity should be obtained for the orbital RT. Possible therapeutic strategies include anti-inflammatory agents, inhibitors of proinflammatory cytokines, and stem cell mobilizers. [36–39]. The efficacy of these strategies on the meibomian gland needs more evidence, since little is known about the pathogenesis of orbital RT on the meibomian gland.

Last, some limitations in this study should be considered. First, the sample size is relatively small due to the difficulty of recruiting appropriate patients. Second, our study did not compare the patients before and after RT. The patients might have meibomian gland dysfunction before RT treatment, which may affect the meibomian gland structure. Only two patients received meibography examination before RT, and one of them is shown in Figure 2. Further perspective study could be designed to confirm our conclusions and test the efficacy of possible preventive strategies.

Overall, our study found prominent damages of the bilateral meibomian gland in patients 12 months after unilateral orbital RT. Such meibomian gland damage might impair ocular surface health, thus more effective protection measures should be taken to minimize this underestimated side effect of orbital radiotherapy.

## Figures and Tables

**Table 1:** Radiotherapy details of patients undergoing RT.

Patient no.	Sex	Age	Diagnosis	RT beam type	RT dosage (Gy)	RT technique	RT area
1	M	79	MALToma	6 MV photons	45	Tomotherapy	L
2	M	47	Eyelid basal cell carcinoma	5 MeV electron	70	Conventional radiotherapy	R
3	F	53	MALToma	8 MeV electrons	30	Conventional radiotherapy	R
4	F	33	MALToma	6 MV photons	45	Tomotherapy	L
5	M	72	MALToma	6 MV photons	50	Tomotherapy	R
6	M	45	Eyelid basal cell carcinoma	7 MeV electrons	50	Conventional radiotherapy	L
7	M	46	MALToma	6 MV photons	45	Tomotherapy	R
8	F	42	Ocular lymphoma	6 MeV electrons	60	Conventional radiotherapy	R
9	M	61	Ocular lymphoma	6 MV photons	40	Tomotherapy	L
10	F	38	MALToma	6 MV photons	40	Tomotherapy	R

M: male; F: female; RT: radiotherapy; MALToma: mucosa-associated lymphoid tissue lymphoma; R: right orbital area; L: left orbital area.

**Table 2:** Comparison of the meibomian gland structure and function between the RT and control group.

	Diseased eyes (*n*=10)	Contralateral eyes (*n*=10)	Normal controls (*n*=10)	*p* ^1^ value	*p* ^2^ value	*p* ^3^ value
Upper MGL	37.79 (12.87, 92.41)	25.54 (15.87, 45.47)	12.63 (6.13, 42.34)	**0.022**	**0.007**	**0.019**
Lower MGL	61.31 (44.67, 87.98)	24.37 (11.94, 49.01)	15.53 (7.65, 45.13)	**0.005**	**≤0.001**	0.089
LLT	40 (23, 100)	68 (12, 100)	81.5 (54, 100)	0.114	**0.007**	0.280
TBUT	3.5 (2, 8)	4.5 (2, 10)	6.5 (5, 10)	**0.041**	**0.002**	**0.019**
Schirmer I test	9 (5, 12)	10.5 (2, 16)	12 (2, 15)	0.280	0.089	0.083
Cornea staining score	1 (0, 3)	0 (0, 4)	0 (0, 1)	0.564	0.075	0.315

All values are described as median (ranges). *p*^1^: diseased eyes vs contralateral eyes, Wilcoxon rank sum test; *p*^2^: diseased eyes vs normal controls, Mann–Whitney test; *p*^3^: contralateral eyes vs normal controls, Mann–Whitney test. *p* values less than 0.05 are considered significant and highlighted in bold. RT: radiotherapy; MGL: meibomian gland loss; LLT: lipid layer thickness; TBUT: tear film breakup time.

**Table 3:** Correlation analysis between the RT dosage and ocular surface parameters (Spearman rank correlation).

	Diseased eyes		Contralateral eyes	
	*r*	*p*	*r*	*p*
RT vs upper MGL	0.285	0.457	−0.207	0.594
RT vs lower MGL	0.913	**0.001**	−0.659	0.054
RT vs LLT	0.080	0.838	0.477	0.194
RT vs TBUT	0.233	0.546	0.538	0.135
RT vs Schirmer I test	0.449	0.226	0.420	0.260
RT vs cornea staining score	0.680	**0.044**	0.609	0.081

*r*: correlation coefficient. *p* values less than 0.05 are considered significant and highlighted in bold. RT: radiotherapy dosage; MGL: meibomian gland loss; LLT: lipid layer thickness; TBUT: tear film breakup time.

## Data Availability

The data used to support the findings of this study are available from the corresponding author upon request.

## Abbreviations

RT: Radiotherapy
MGL: Meibomian gland loss
LLT: Lipid layer thickness
TBUT: Tear film breakup time
OSDI: Ocular surface disease index
ICU: Interferometry color units.
